# Textbook Outcomes of Totally Robotic Versus Totally Laparoscopic Pancreaticoduodenectomy for Periampullary Neoplasm: A Propensity Score-Matched Cohort Study

**DOI:** 10.3390/jcm14186687

**Published:** 2025-09-22

**Authors:** Boram Lee, Ho-Seong Han, Yoo-Seok Yoon, Jun Suh Lee

**Affiliations:** Department of Surgery, Seoul National University Bundang Hospital, Seoul National University College of Medicine, Seongnam-si 13605, Republic of Korea; boramsnubhgs@gmail.com (B.L.); yoonys@snubh.org (Y.-S.Y.); rudestock@gmail.com (J.S.L.)

**Keywords:** pancreaticoduodenectomy, laparoscopy, robotic surgical procedures, periampullary neoplasms, minimally invasive surgical procedures

## Abstract

**Background/Objectives**: Textbook outcome (TO) is a composite quality measure in surgery, but few studies have compared TO between robotic pancreaticoduodenectomy (RPD) and laparoscopic pancreaticoduodenectomy (LPD). This study aimed to evaluate and compare TO following RPD and LPD for periampullary neoplasms. **Methods**: We retrospectively analyzed 322 patients who underwent minimally invasive PD between 2010 and 2023 (RPD, *n* = 60; LPD, *n* = 262). LPD was first introduced in 2004, but only cases performed since 2010 were included, while RPD has been performed since 2019. Propensity score matching (1:2) yielded 48 RPD and 96 LPD patients. TO was defined as the absence of pancreatic fistula, bile leak, post-pancreatectomy hemorrhage, severe complications (Clavien-Dindo ≥ III), readmission, and in-hospital or 30-day mortality. **Results**: In the entire cohort, 240 of 322 patients (74.5%) achieved TO. After matching, TO rates were 64.6% in RPD and 76.9% in LPD (*p* = 0.656). Perioperative outcomes, including operative time, blood loss, transfusion, hospital stay, and major complications, were comparable, although RPD showed a higher incidence of hemorrhage (*p* = 0.032). Multivariate analysis identified body mass index < 25 kg/m^2^ as an independent predictor of achieving TO (OR 3.13, *p* = 0.008). **Conclusions**: RPD and LPD achieved comparable textbook outcomes in periampullary surgery. Both approaches are feasible when performed by experienced surgeons, but larger studies with long-term follow-up are needed to validate these findings.

## 1. Introduction

Pancreaticoduodenectomy (PD) is the major surgical treatment for periampullary neoplasms. Historically, this procedure has been challenging because of its technical complexity and significant risk of complications [1,2]. Advances in surgical techniques have led to the development of minimally invasive surgery (MIS) such as laparoscopic pancreaticoduodenectomy (LPD) and robotic pancreaticoduodenectomy (RPD). These methods aim to improve patient outcomes by reducing surgical trauma and promoting faster recovery while maintaining oncological efficacy [3,4,5].

The integration of MIS into complex abdominal surgery has been gradual because of concerns regarding its technical feasibility and patient safety. For instance, the robotic platform offers several advantages, including superior three-dimensinal visualization, increased range of instrument movement, and improved ergonomics for the surgeon [6,7]. These features are expected to improve the post-operative outcomes. Nonetheless, when new surgical methods such as RPD are introduced, consideration of patient safety is important to ensure that technological advances do not compromise patient safety.

One way to comprehensively assess surgical quality and outcomes is to use the “textbook outcome (TO)” concept. TO is a multidimensional measure that reflects the “ideal” surgical result based on specific criteria [8]. TO has been associated with improved patient survival in surgeries such as colorectal cancer, liver metastasis, lung transplantation, and head and neck cancer [9,10,11]. It serves as a valuable benchmarking tool for assessing the quality of care provided to patients undergoing various surgical interventions, highlighting areas for improvement and enhancing post-operative prognosis. Despite its importance, there has been a lack of research on TO in pancreatic surgery, but it has recently been established through international surgery among experts [12]. PD is one of the most complex abdominal operations, and its minimally invasive approaches, including LPD and RPD, are technically demanding with steep learning curves. With the increasing adoption of minimally invasive techniques worldwide, evaluating TO in this setting is essential to ensure both safety and oncologic adequacy. TO was defined as the absence of post-operative pancreatic fistula, bile leak, post-pancreatectomy hemorrhage, severe complications, readmission, or in-hospital mortality. Unfortunately, no studies have yet utilized TO to compare short-term surgical outcomes between RPD and LPD.

With the increasing adoption of robotic systems in pancreatic surgery and the increasing importance of outcome measures, our study aimed to compare the perioperative outcomes (based on TO) between RPD and LPD in the treatment of periampullary tumors. To ensure comparability between the two groups and to minimize the selection bias, propensity score matching (PSM) was applied.

## 2. Materials and Methods

### 2.1. Patients

This retrospective cohort study was conducted at a single center at Seoul National University Bundang Hospital, Seoul National University College of Medicine, Korea. We reviewed retrospectively collected data of patients who underwent RPD or LPD for periampullary tumors between January 2010 and December 2023. Laparoscopic PD was first introduced at our center in 2004, but only a very small number of cases were performed between 2004 and 2010; these early cases were excluded. Therefore, the present cohort of 322 patients essentially represents all consecutive MIPDs performed since 2010, along with all robotic PDs since their introduction in 2019. At our center, approximately 100–150 open PDs are performed annually. The choice between open and minimally invasive PD was based on tumor resectability, patient condition, and surgeon discretion. In the early period of our MIPD program, selection was more conservative, and patients with borderline resectability, neoadjuvant therapy, or vascular involvement were more frequently treated with open PD. With increasing experience, the indications for MIPD have expanded.

All minimally invasive procedures were performed by four experienced faculty surgeons in the hepatobiliary and pancreatic division. All had surpassed the recognized learning curve for MIPD, having performed >50 laparoscopic PDs or >30 robotic PDs before the study period. This ensured that the procedures included in this analysis were performed after the learning curve had been achieved.

Patients who underwent combined resection of other organs were excluded from this study. Patients were given the option to undergo RPD or LPD, and each patient selected the type of surgery. This study was approved by the Institutional Review Board (IRB) of Seoul National University Bundang Hospital (B-2407-912-101). All procedures followed were in accordance with the ethical standards of the responsible committee on human experimentation and the Helsinki Declaration of 1975, as revised in 2000. As this was a retrospective study and the data were anonymized, the requirement for informed consent was waived by the IRB.

### 2.2. Surgical Procedures

**Figure 1:**
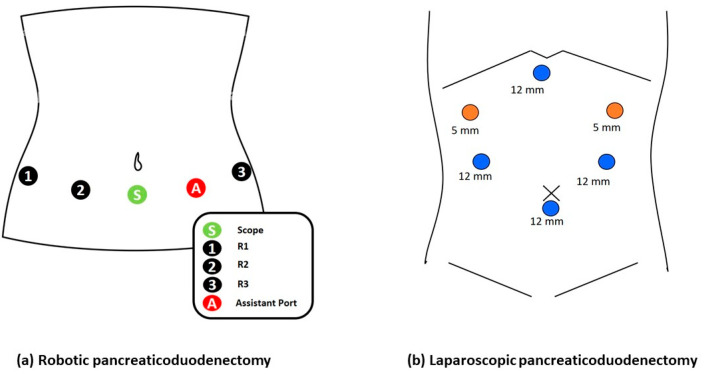
Trocar placement for robotic and laparoscopic pancreaticoduodenectomy. (**a**) Robotic pancreaticoduodenectomy: trocar positions include the scope port (S) centrally, robotic arms (R1, R2, R3) laterally, and an assistant port (A) on the patient’s left side. (**b**) Laparoscopic pancreaticoduodenectomy: trocar positions include three 12 mm ports for instruments (blue circles) and two 5 mm ports for additional instruments (orange circles), arranged around the surgical site.

Totally robotic pancreaticoduodenectomy (RPD): The RPD was first initiated at our institution in January 2018. We performed using the da Vinci Xi system (Intuitive Surgical, Sunnyvale, CA, USA). For the procedure, patients were positioned in the lithotomy and reversed Trendelenburg positions with a slight elevation on the right side. The assistant stood between the patient’s legs. The trocar placement is illustrated in Figure 1a. Our approach to RPD followed standardized operative strategies that have been described in previous studies [13,14]. All reconstruction methods were performed using an intracorporeal anastomosis.

Totally laparoscopic pancreaticoduodenectomy (LPD): The LPD was first initiated at our institution in January 2010. Similar to RPD, patients undergoing LPD are placed in the lithotomy and reverse Trendelenburg positions. The trocar placement is shown in Figure 1b. We adhered to standardized operative strategies for LPD, as previously described. Pancreaticojejunostomy (PJ) and choledochojejunostomy (CJ) are performed using intracorporeal anastomosis, whereas the duodenojejunstomy (DJ) is completed extracorporeally.

### 2.3. Variables

The demographic and clinical data collected included age, sex, body mass index (BMI), pre-existing conditions such as hypertension (HTN) and diabetes mellitus (DM), American Society of Anesthesiologists (ASA) score, Eastern Cooperative Oncology Group (ECOG) performance status, previous abdominal operations, incidental detection of the tumor, weight loss, jaundice, biliary drainage, tumor location, tumor markers (CEA, CA 19-9), tumor size, and neoadjuvant chemotherapy.

### 2.4. Definition of Textbook Outcomes

TO was defined as the simultaneous fulfillment of all of the following criteria: (a) absence of post-operative pancreatic fistula (POPF), (b) absence of bile leak, (c) absence of post-pancreatectomy hemorrhage (all grade B/C according to ISGPS or ISGLS) [15,16] (d) absence of severe complications severe complications (Clavien-Dindo grade ≥ III) [17], (e) absence of readmission within 30 days after discharge, and in-hospital or 30-day mortality, as described in previous studies [12] and in the recent international consensus on textbook outcome and Ideal Outcome in pancreatic surgery [18].

### 2.5. Statistical Analysis

**Figure 2:**
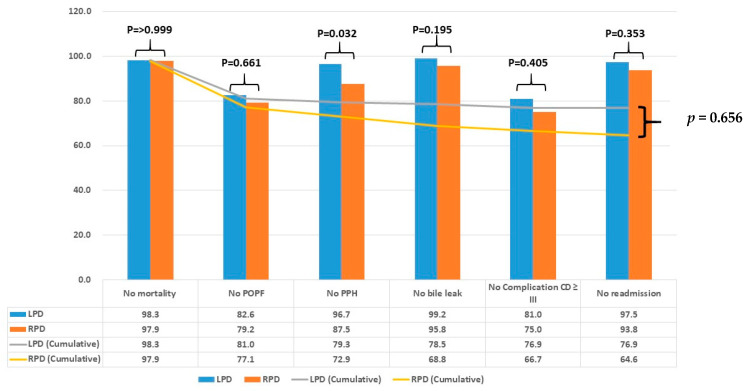
Comparison of textbook outcomes between laparoscopic pancreaticoduodenectomy (LPD) and robotic pancreaticoduodenectomy (RPD).

PSM was used to balance the baseline characteristics between the two groups. Variables that showed significant imbalances in the unmatched cohort, namely sex and ASA score, were included in the propensity score model. Matching was conducted at a 1:2 ratio using the nearest-neighbor method without replacement. The analysis compared TO, as well as other perioperative outcomes such as operation time, estimated blood loss (EBL), transfusion rates, and length of hospital stay. Chi-square or Fisher’s exact tests were used for categorical variables, and *t*-tests or Mann–Whitney U tests were used for continuous variables, as appropriate. Statistical significance was set at *p* < 0.05. Incidences of each individual outcome criterion contributing to TO were compared between the two groups using cross-tabulation with Chi-square or Fisher’s exact tests, as appropriate. Cumulative trends in TO achievement (Figure 2) were generated descriptively by sequentially plotting the proportion of patients fulfilling each outcome criterion. No formal statistical trend test or regression analysis was applied. Multivariate logistic regression was used to identify the factors independently associated with TO achievement. Univariable and multivariable logistic regression analyses were performed on the propensity score-matched cohort to identify independent factors associated with achieving TO. Odds ratios (ORs) with 95% confidence intervals (CIs) were calculated.

## 3. Results

### 3.1. Baseline Characteristics

In our study evaluating the demographic of robotic pancreaticoduodenectomy (RPD) and laparoscopic pancreaticoduodenectomy (LPD), we initially analyzed 322 patients; 60 underwent RPD and 262 underwent LPD (Table 1). After employing a 1:2 PSM to reduce potential biases and ensure comparability, our focus shifted to 48 RPD and 96 LPD patients. In our study comparing RPD and LPD, demographic analysis revealed no significant differences between the groups in most measured parameters, confirming the effective PSM. Specifically, in the matched cohorts, the proportion of males was slightly higher in the RPD group (78.0%) than in the LPD group (71.0%), although this difference was not statistically significant (*p* = 0.78). Age and body mass index (BMI) between the two groups were closely matched, with mean ages of 64.8 ± 12.7 years for RPD and 65.2 ± 11.5 years for LPD, and mean BMIs of 24.4 ± 3.6 kg/m^2^ for RPD and 24.2 ± 3.0 kg/m^2^ for LPD, both showing no significant differences (*p* = 0.521 for age and *p* = 0.761 for BMI). Clinical comorbidities, such as hypertension and diabetes mellitus, also showed no significant differences between the RPD and LPD groups in the matched cohorts. ASA scores and ECOG performance statuses were similarly distributed, indicating comparable baseline health and functional status. The primary tumor locations and tumor markers, CEA and CA 19-9, were similarly well balanced between the groups. Importantly, the proportion of malignant tumors was comparable in the matched cohort (RPD 83.3% vs. LPD 82.3%, *p* = 0.880)

### 3.2. Short-Term Outcomes

In evaluating the operative outcomes between RPD and LDP, our study found no significant differences in most surgical metrics and complications, indicating a comparable surgical performance between the two techniques (Table 2). Operation time for RPD averaged 543.6 ± 117.4 min compared to 444.2 ± 135.7 min for LPD, showing a trend towards longer duration for RPD, although this difference was not statistically significant (*p* = 0.372). Estimated blood loss was similar between the groups with RPD at 401.1 ± 383.7 mL and LPD at 362.8 ± 352.7 mL (*p* = 0.615). The transfusion rate was identical in both groups at 8.3% (*p* > 0.999). Regarding the condition of the pancreas, a slightly higher percentage of patients in the RPD group had a soft pancreas (72.9% in RPD vs. 69.8% in LPD, *p* = 0.892), and the pancreatic duct size was comparable between the groups (2.58 ± 1.89 mm for RPD vs. 2.82 ± 1.67 mm for LPD, *p* = 0.768). In terms of complications classified by the Clavien-Dindo grade, RPD had a higher incidence of grade ≥ III complications, although this difference was not significant (*p* = 0.136). The occurrence of clinically relevant post-operative pancreatic fistula (CR-POPF) was also similar (20.8% in RPD vs. 17.8% in LPD, *p* = 0.176), and there was no significant difference in the average hospital stay (12.3 ± 11.4 days for RPD vs. 11.6 ± 5.7 days for LPD, *p* = 0.281). Importantly, as a short-term oncologic indicator, the R0 resection rate was comparable between groups (95.0% in RPD vs. 94.9% in LPD, *p* = 0.981).

### 3.3. Textbook Outcomes

Figure 2 shows a comparison of the TO rates for RPD and LPD. Both techniques exhibited high survival rates, with no statistically significant difference in mortality (LPD 98.3% vs. RPD 97.9%, *p* > 0.999). Similarly, the rate for no readmission was high in both groups, with LPD showing slightly better outcomes (LPD 97.5% vs. RPD 93.8%, *p* = 0.353). For POPF, there was no significant difference between the groups (LPD 82.6% vs. RPD 79.2%, *p* = 0.661). However, the incidence of post-pancreatectomy hemorrhage (PPH) was significantly different, with LPD patients experiencing fewer events than RPD patients (LPD 96.7% vs. RPD 87.5%, *p* = 0.032). No bile leak rates were high in either group, with no significant difference noted (LPD 99.2% vs. RPD 95.8%, *p* = 0.195). In terms of severe complications (Clavien-Dindo grade ≥ III), RPD patients experienced a higher rate of complications, although the difference was not statistically significant (LPD 81.0% vs. RPD 75.0%, *p* = 0.405). The overall proportions of patients who achieved TO were 76.9% in LPD and 64.6% in the RPD. Across the entire cohort of 322 patients, 240 (74.5%) achieved TO. The cumulative outcomes across all parameters indicated no significant overall difference between RPD and LPD (*p* = 0.656), suggesting that both techniques are viable options for pancreaticoduodenectomy, each with specific strengths and challenges.

The bar graph shows the percentage of patients achieving specific textbook outcomes: no mortality, no post-operative pancreatic fistula (POPF), no post-pancreatectomy hemorrhage (PPH), no bile leak, no complications classified as Clavien-Dindo grade ≥ III, and no readmission. The cumulative textbook outcome rates are also presented. LPD is represented by blue bars, and RPD by orange bars. Cumulative outcome trends are depicted by gray and yellow lines for LPD and RPD, respectively. *p*-values indicate the statistical significance of differences between LPD and RPD for each outcome measure. The overall comparison of textbook outcomes showed no significant difference between the two groups (*p* = 0.656). Comparisons of incidence between groups were performed using Chi-square or Fisher’s exact tests, as appropriate. The cumulative trends displayed in the line graphs are descriptive visualizations of sequential achievement of each TO component; no formal trend test was applied.

### 3.4. Multivariate Analysis

Table 3 presents the multivariate analysis of the variables associated with TO achievement after PD. Multiple variables were considered in the analysis, including the method of surgery (LPD vs. RPD), sex, age, previous abdominal surgery, ASA score, BMI, preoperative CA 19-9 levels, pre-operative diabetes mellitus, pre-operative jaundice, pancreatic duct size, pancreatic hardness, operative time, blood loss, intraoperative transfusion, and pathology. Significant findings included BMI < 25 kg/m^2^, which showed a higher likelihood of achieving TO (OR 3.134, *p* = 0.008).

## 4. Discussion

In this study, we evaluated and compared TO between RPD and LPD in patients with periampullary neoplasms. When introducing a new surgical technique, it is crucial to assess its stability and effectiveness. In this context, TO serves as a comprehensive measure to evaluate the quality and reliability of surgical techniques. Our findings suggest that both RPD and LPD are viable surgical options, with comparable outcomes for most measured parameters.

Before matching, the proportion of male patients was significantly higher in the RPD group than in the LPD group. This predominance of male patients may reflect the epidemiologic characteristics of periampullary and pancreatic neoplasms, which are more frequently observed in men in Korea [19]. After propensity score matching, demographic analysis showed no significant differences between the RPD and LPD groups, indicating effective adjustment. PSM was implemented to reduce potential biases, particularly because RPD started relatively recently compared with LPD, and the indications for RPD have been applied more strictly. This balance in the baseline characteristics strengthens the validity of our comparative analysis. It is also noteworthy that the rate of NAT in our cohort was low. At our institution, NAT has been generally reserved for patients with borderline resectable or locally advanced periampullary tumors, whereas patients with clearly resectable disease proceeded directly to surgery. During the early period of our MIPD program, patients who had received NAT were more often treated with open PD, reflecting the cautious patient selection inherent to the learning phase. However, as our institutional experience accumulated and surgical proficiency improved, the indications for MIPD have expanded, and more patients undergoing NAT are now considered candidates for minimally invasive approaches. Although laparoscopic and robotic pancreatectomy have recently shown promising results in patients who received NAT [20,21], the current evidence remains limited, and further data are needed to establish their safety in this setting.

In terms of short-term operative outcomes, there were no significant differences in operation time, EBL, transfusion rates, or length of hospital stay between the RPD and LPD groups. Although RPD was started relatively recently compared with LPD at our institution, the short-term outcomes of the two modalities were similar. This suggests that RPD has the advantage of a short learning curve. The learning curve for RPD appears to be shorter than that for LPD based on the provided research data. Studies on RPD show that proficiency benchmarks were achieved in approximately 30 cases [22,23,24]. In contrast, LPD studies suggest that technical competency was reached after approximately 52 procedures, with mastery achieved after 94 cases [22,23,24]. This indicates that the learning curve for RPD may indeed be shorter compared to that for LPD, making robotic-assisted procedures a promising option for minimally invasive pancreatic surgery.

However, the incidence of PPH was significantly higher in the RPD group compared to the LPD group. This difference may be attributed to immaturity before the learning curve is reached. Notably, there was no PPH after the middle of the RPD period, indicating that experience and proficiency significantly reduced this complication. Despite this, the overall rate of TO achievement was not significantly different between the groups, indicating that both techniques were capable of delivering optimal surgical outcomes.

Multivariate analysis identified that a BMI of less than 25 kg/m^2^ was associated with a higher likelihood of achieving a TO. Chao et al. suggested several key reasons why a lower BMI (<25 kg/m^2^) is beneficial for achieving favorable outcomes in RPD [25]. A lower BMI typically results in less visceral fat, which facilitates easier access and visualization of anatomical structures during surgery. Obesity is associated with increased post-operative complications such as wound infection, anastomotic leak, and respiratory issues. In contrast, patients with a low BMI have a decreased risk of complications, which improves their overall surgical outcomes [26]. These findings highlight the importance of patient selection and pre-operative optimization to achieve optimal surgical results in minimally invasive pancreatic surgery.

The absence of preoperative jaundice and a firm/hard pancreas were also associated with better outcomes in univariate analysis but were not identified as significant factors in multivariate analysis. This is probably because there were no significant differences in these factors in the patient group indicated for MIS. Given these advantages, it is crucial to carefully select patients in the early stages of RPD or LPD to maximize the likelihood of achieving TO. Factors such as low BMI and a firm/hard pancreas should be prioritized when selecting patients for these procedures. This involves thorough preoperative assessment and optimization, particularly in patients with a higher BMI. Implementing tailored protocols and ensuring meticulous patient selection can significantly enhance surgical success rates and post-operative recovery rates.

Our study had several limitations, including its retrospective design and the single-center setting, which may limit the generalizability of our findings. While the short-term results after surgery are important, the results of oncological stability after surgery are also crucial. However, this study did not include long-term oncological outcomes because robotic surgery has only started relatively recently. Moreover, although the TO rate was numerically lower in the RPD group than in the LPD group, this difference did not reach statistical significance. This study may therefore have been underpowered to detect clinically meaningful differences, and the findings should be interpreted with caution. Further prospective studies and long-term follow-up are warranted to confirm these results and to assess the impact on long-term oncological outcomes.

## 5. Conclusions

In conclusion, RPD and LPD can achieve similar TO for PD in the context of periam-pullary neoplasms. While RPD offers certain advantages, such as enhanced visualization and ergonomics, careful patient selection and management of intraoperative challenges are essential to optimize outcomes. This study contributes to the growing body of evidence supporting the use of minimally invasive techniques in complex abdominal surgery, emphasizing the need for careful patient selection and management of intraoperative challenges to optimize outcomes.

## Figures and Tables

**Table 1:** Baseline patient and disease characteristics.

		Entire Cohort			Matched Cohort		
		RPD (*n* = 60)	LPD (*n* = 262)	*p*	RPD (*n* = 48)	LPD (*n* = 96)	*p* Value
Male, *n* (%)	57 (76.7%)	178 (68.0%)	0.038	39 (78.0%)	68 (71.0%)	0.78	
Age, mean [SD], years	65.3 ± 11.5	64.4 ± 13.1	0.369	64.8 ± 12.7	65.2 ± 11.5	0.521	
BMI, mean [SD], Kg/m^2^	24.3 ± 3.0	24.1 ± 4.2	0.595	24.4 ± 3.6	24.2 ± 3.0	0.761	
HTN, *n* (%)	20 (33.3%)	125 (47.7%)	0.303	19 (38.0%)	50 (52.1%)	0.113	
DM, *n* (%)	19 (31.7%)	56 (21.5%)	0.211	17 (34.0%)	21 (21.8%)	0.331	
ASA, *n* (%)			0.677			0.297	
1	15 (25.0%)	54 (20.7%)		14 (28.0%)	18 (18.8%)		
2	40 (66.7%)	172 (65.6%)		31 (62.0%)	65 (67.7%)		
3	5 (8.3%)	36 (13.8%)		5 (10.5%)	13 (13.5%)		
ECOG, *n* (%)			0.472			0.403	
0	52 (86.7%)	210 (80.5%)		42 (84.0%)	87 (90.6%)		
1	8 (13.3%)	51 (19.5%)		8 (16.0%)	9 (9.4%)		
Previous abdominal operation hx. *n* (%)	17 (28.3%)	88 (33.6%)	0.187	11 (25.0%)	37 (38.5%)	0.119	
Incidental detection, *n* (%)	6 (10.0%)	36 (13.7%)	0.701	6 (13.6%)	17 (17.7%)	0.805	
Weight loss, *n* (%)	25 (41.7%)	107 (40.8%)	0.881	17 (38.6%)	44 (45.8%)	0.458	
Jaundice, *n* (%)	21 (35.0%)	87 (33.2%)	>0.999	16 (32.0%)	37 (38.5%)	0.518	
Biliary drainage	26 (43.3%)	134 (51.1%)	0.593	26 (52.0%)	57 (59.3%)	0.247	
Tumor location			0.306			0.405	
Pancreas	24 (40.0%)	82 (31.3%)		19 (38.0%)	21 (21.9%)		
Common bile duct	24 (40.0%)	106 (40.5%)		19 (38.0%)	38 (39.6%)		
AoV	12 (20.2%)	74 (28.2%)		12 (24.0%)	37 (38.5%)		
Malignancy, *n* (%)		50 (83.8)	214 (81.7)	0.791	40 (83.3)	79 (82.3)	0.880
CEA, mean [SD]	4.8 ± 14.1	4.3 ± 21.5	0.883	3.7 ± 2.7	4.3 ± 23.5	0.782	
CA 19-9, mean [SD]	369.3 ± 127.1	207.5 ± 755.9	0.638	341.5 ± 449.5	212.7 ± 819.2	0.731	
Tumor size (cm), mean [SD]	2.5 ± 1.0	3.04 ± 1.9	0.218	2.4 ± 1.1	2.9 ± 1.4	0.463	
Neoadjuvant CTx, *n* (%)	0	1 (0.4%)	>0.999	0	0	>0.999	

RPD: Robotic Pancreaticoduodenectomy; LPD: Laparoscopic Pancreaticoduodenectomy; SD: Standard Deviation; BMI: Body Mass Index; HTN: Hypertension; DM: Diabetes Mellitus; ASA: American Society of Anesthesiologists; ECOG: Eastern Cooperative Oncology Group; Hx: History; CEA: Carcinoembryonic Antigen; CA 19-9: Carbohydrate Antigen 19-9; CTx: Chemotherapy; AoV = Ampulla of Vater. All variables are presented as the mean and standard deviation or *n* (%) of patients.

**Table 2:** Operative outcome.

	Matched Cohort		
	RPD (*n* = 48)	LPD (*n* = 96)	*p* Value
Op time, mean [SD], min	543.6 ± 117.4	444.2 ± 135.7	0.372
EBL, mean [SD], mL	401.1 ± 383.7	362.8 ± 352.7	0.615
Transfusion, *n* (%)	4 (8.3%)	10 (8.3%)	>0.999
Soft pancreas	35 (72.9%)	67 (69.8%)	0.892
P-duct size (mm), mean [SD]	2.58 ± 1.89	2.82 ± 1.67	0.768
C-D grade, *n* (%)			0.136
III	10 (20.8%)	15 (15.6%)	
IV	2 (4.2%)	1 (1.0%)	
V	0	2 (2.1%)	
CR-POPF, *n* (%)	10 (20.8%)	17 (17.8%)	0.176
Hospital days, mean [SD], days	12.3 ± 11.4	11.6 ± 5.7	0.281
Margin status (R0), *n* (%)	38 (95.0)	75 (94.9)	0.981

RPD: Robotic Pancreaticoduodenectomy; LPD: Laparoscopic Pancreaticoduodenectomy; SD: Standard Deviation; Op: Operation; EBL: Estimated Blood Loss; P-duct: Pancreatic Duct; C-D grade: Clavien-Dindo Grade; CR-POPF: Clinically Relevant Post-Operative Pancreatic Fistula.

**Table 3:** Multivariate analysis of variables associated with textbook outcome after pancreaticoduodenectomy.

	Univariate Analysis		Multivariate Analysis	
Variable	Odds Ratio (95% CI)	*p* Value	Odds Ratio (95% CI)	*p* Value
**Operation method (*n*/%)**		0.496		
LPD	Ref			
RPD	0.727 (0.289–1.824)			
**Sex (*n*/%)**		0.577		
Male	Ref			
Female	1.246 (0.576–2.696)			
**Age (Years)**		0.696		
<70 years	Ref			
>70 years	1.166 (0.538–2.526)			
**Previous abdominal surgery (*n*/%)**		0.635		
Yes	Ref			
NO	0.820 (0.360–1.866)			
**ASA score (*n*/%)**				
1	Ref	0.345		
2 or 3	1.594 (0.605–4.197)			
**BMI (kg/m^2^)**		0.002		0.008
<25 kg/m^2^	2.302 (1.139–4.656)		3.134 (1.349–7.282)	
>25 kg/m^2^	Ref		Ref	
**Preoperative CA 19-9**		0.266		
<250	Ref			
>250	0.522 (0.166–1.642)			
**Preoperative DM (*n*/%)**		0.393		
Yes	Ref			
No	0.655 (0.248–1.729)			
**Preoperative jaundice (*n*/%)**		0.037		
Yes	Ref			
No	0.411 (0.179–0.946)			
**Pancreas duct size (mm)**		0.017		
<3 mm	0.391 (0.269–0.594)			
>3 mm	Ref			
**Pancreas hardness (*n*/%)**		<0.001		
Soft	0.193 (0.280–0.825)			
Firm/Hard	Ref			
**Operative time (min)**		0.074		
<300 min	Ref			
>300 min	0.448 (0.186–1.081)			
**Blood loss (mL)**		0.192		
<500 mL	Ref			
>500 mL	0.546 (0.238–1.334)			
**Inatraoperative transfusion (*n*/%)**		0.001		
Yes	Ref			
No	0.283 (0.335–0.609)			
**Pathology (*n*/%)**		0.838		
Pancreatic cancer	Ref			
Non-pancreatic cancer	0.320 (0.541–0.646)			

RPD: Robotic Pancreaticoduodenectomy; LPD: Laparoscopic Pancreaticoduodenectomy; SD: Standard Deviation; BMI: Body Mass Index; HTN: Hypertension; DM: Diabetes Mellitus; ASA: American Society of Anesthesiologists; ECOG: Eastern Cooperative Oncology Group; Hx: History; CEA: Carcinoembryonic Antigen; CA 19-9: Carbohydrate Antigen 19-9; CTx: Chemotherapy; CI: Confidence Interval.

## Data Availability

The datasets used and analyzed during the current study are available from the corresponding author upon reasonable request.
